# Efficacy of progesterone for moderate to severe traumatic brain injury: a meta-analysis of randomized clinical trials

**DOI:** 10.1038/srep13442

**Published:** 2015-08-25

**Authors:** Chao Lin, Hongquan He, Zheng Li, Yinglong Liu, Honglu Chao, Jing Ji, Ning Liu

**Affiliations:** 1 Department of Neurosurgery, the First Affiliated Hospital of Nanjing Medical University, 300 Guangzhou Road, Nanjing, Jiangsu, 210029 China; 2 Department of Neurosurgery, Gaochun Hospital, Nanjing, Jiangsu Province China

## Abstract

Progesterone has been shown to have neuroprotective effects in multiple animal models of brain injury, whereas the efficacy and safety in patients with traumatic brain injury (TBI) remains contentious. Here, a total of seven randomized controlled trials (RCTs) with 2492 participants were included to perform this meta-analysis. Compared with placebo, there was no significant decrease to be found in the rate of death or vegetative state for patients with acute TBI (RR = 0.88, 95%CI = 0.70, 1.09, p = 0.24). Furthermore, progesterone was not associated with good recovery in comparison with placebo (RR = 1.00, 95%CI = 0.88, 1.14, p = 0.95). Together, our study suggested that progesterone did not improve outcomes over placebo in the treatment of acute TBI.

Traumatic brain injury (TBI) is a leading cause of death and disability and without effective treatment in children and young adults^1^,^2^,^3^. Despite improvement in outcome following brain injury in recent years, large numbers of patients remain disabled and dependent^2^,^4^. Previous animal studies suggested that progesterone could extenuate neural damage effectively by reducing free radicals, inflammatory cytokines, excitotoxicity, apoptosis, and vasogenic edema in the model of neurologic injury^5^,^6^,^7^. However, the relevant clinical trials of progesterone demonstrated different clinical benefits and discrepant conclusions for the treatment of patients with acute TBI^8^,^9^. Treatment recommendations may be misleading according to the results of any individual trial. Based on available data, we performed a meta-analysis of randomized clinical trials to compare progesterone with placebo for the treatment of patients with severe or moderate acute TBI. The overall evaluation was performed to accurately detail the efficacy and safety of progesterone.

## Results

### Study selection and characteristics

**Figure 1:**
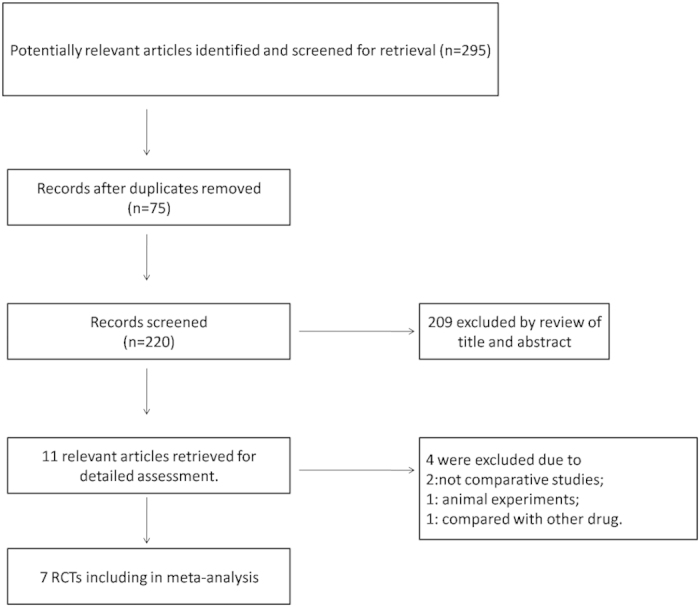
Flow chart of the selection process used for the randomized controlled trials.

The detailed process of selection is shown in Fig. 1. In total, 295 potential studies were identified with a systematic search of databases. Seventy-five records were excluded as duplicates. We removed 209 apparently unsuitable articles including reviews, case reports and animal experiments after browsing titles and abstracts. The remaining studies were screened and assessed in detail by reviewing full texts. Four articles were excluded due to the following reasons: two excluded studies were not comparative trials, 1 study was an animal experiment, and 1 study involved comparison with another drug. Thus, 7 studies meeting our inclusion criteria were selected in this meta-analysis.

These seven studies, with 2492 total participants (Sample size, from 40 to 1179), compared progesterone with placebo in the treatment of acute TBI. Of these, only patients with severe TBI (Glasgow Coma Scale (GCS) ≤8) were included in five studies^8^,^9^,^10^,^11^,^12^, and in another 2 studies patients were recruited with moderate-to-severe TBI (GCS ≤12)^13^,^14^. All female patients were excluded in one included study^12^. The primary characteristics and quality assessments of the included RCTs are summarized in Table 1 and 2, respectively.

### Meta-analysis outcomes

#### Death or vegetative state

**Figure 2:**
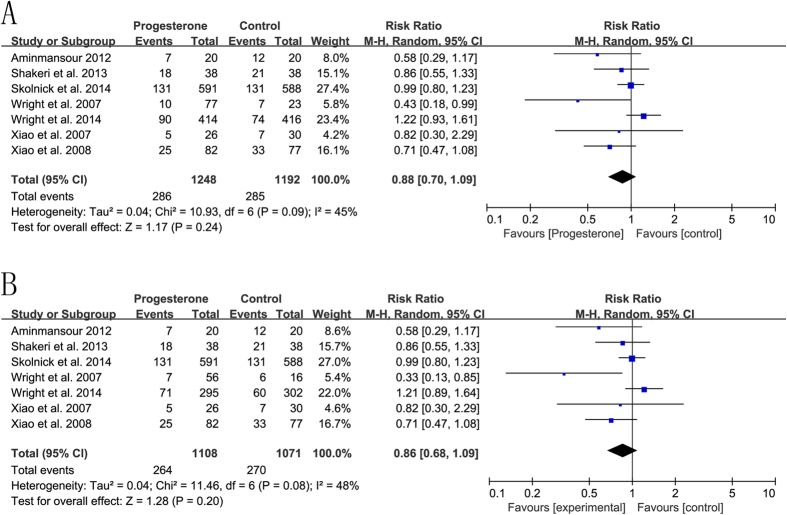
The efficacy of progesterone in reducing the rate of death or vegetative state in comparison to placebo. (**A**) acute traumatic brain injury; (**B**) acute severe traumatic brain injury.

The meta-analysis of seven RCTs with a random-effects model demonstrated that progesterone did not significantly reduce the rate of death or vegetative state in patients with acute TBI between the two groups (RR = 0.88, 95%CI = 0.70, 1.09, p = 0.24, I^2^ = 45%) (Fig. 2A). Subgroup meta-analysis was performed (Table 3). Due to the limited number of available studies, meta-regression was not pursued further. A similar result was observed in patients with severe TBI (RR = 0.86, 95%CI = 0.68, 1.09, p = 0.20, I^2^ = 48%) (Fig. 2B). The sensitivity analysis was performed to examine the influence of different models on the pooled estimates. There were no significant changes to be found with a fixed-effects model (RR = 0.97, 95%CI = 0.84, 1.11, p = 0.65; RR = 0.95, 95%CI = 0.82, 1.10, p = 0.50).

### Good recovery

**Figure 3:**
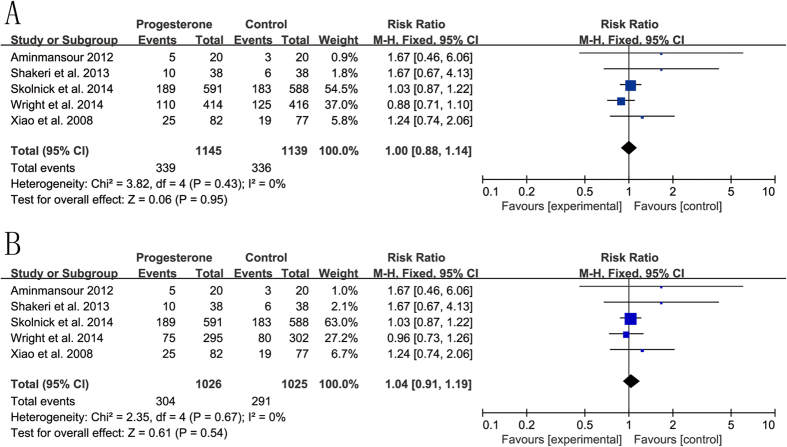
The efficacy of progesterone in improving outcomes (good recovery) in comparison with placebo. (**A**) acute traumatic brain injury; (**B**) acute severe traumatic brain injury.

In total, five of the included RCTs had an assessment of good recovery (GOS = 5) at the end of follow-up. No significant heterogeneity was observed in TBI (I^2^ = 0%). Compared with placebo, the combined data using a fixed-effects model did not show that progesterone significantly increased the rate of good recovery (RR = 1.00, 95%CI = 0.88, 1.14, p = 0.95) (Fig. 3A). There was also no evidence to indicate that progesterone could improve the outcome for a good recovery in severe TBI (RR = 1.04, 95%CI = 0.91, 1.19, p = 0.54, I^2^ = 0%) (Fig. 3B).

### Adverse events

**Figure 4:**
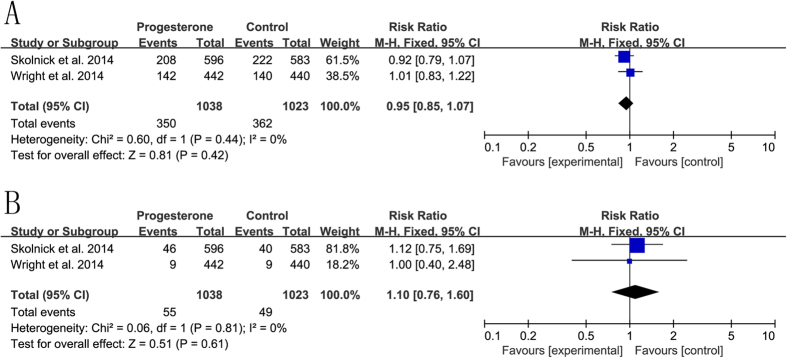
Safety of progesterone in the treatment of traumatic brain injury. (**A**) pneumonia; (**B**) sepsis.

Two studies were included in the meta-analysis of adverse events. A fixed-effects model was used according to heterogeneity. There were no statistically significant differences in pneumonia or sepsis between the two groups (RR = 0.95, 95%CI = 0.85, 1.07, p = 0.42, I^2^ = 0%; RR = 1.10, 95%CI = 0.76, 1.60, p = 0.61, I^2^ = 0%) (Fig. 4).

### Publication bias

There was no evidence of publication bias (Begg’s test, P = 0.90; Egg’s test, P = 0.059).

## Discussion

The pathophysiology of acute TBI is a complex, interwoven and multifactorial process, which includes primary and secondary injury^15^,^16^. TBI-induced secondary injury has been considered to be a potential target for therapeutic intervention involving reduction and prevention of inflammation, calcium flux, oxidative stress, necrosis, and apoptosis^17^,^18^. Based on the efficacy and safety in animal models, progesterone has been regarded to be a potent candidate for the treatment of TBI^19^,^20^,^21^,^22^. However, the relevant clinical trials of progesterone came to inconsistent conclusions^10^,^12^,^14^. The previous review of progesterone for the treatment of TBI included only three small-scale and low-quality studies^23^. In this current study, we selected 7 relevant RCTs including 2492 patients (progesterone: 1276 cases, placebo: 1216 cases) hospitalized for acute TBI to assess the efficacy of progesterone therapy on the Glasgow Outcome Scale (GOS) score and for adverse events.

Some previous clinical studies demonstrated that progesterone was a neuroprotective agent and improved outcomes for patients with acute severe TBI^8^,^10^. However, we found no significant difference between the progesterone-treated group and the placebo group in the rate of death or vegetative state. Moreover, our results showed that progesterone was not associated with good recovery at the end of the follow-up period. To date, various drugs have been investigated in clinical trials, yet none has been proven to reduce mortality significantly at the confirmatory stage^24^,^25^,^26^,^27^. The trauma of individual patients could not be controlled well in comparison with the animal model. The heterogeneity and variability of TBI may be one of the important reasons^14^,^28^. This classification scheme of patients may be relatively insensitive using the Glasgow Coma Scale (GCS) or the Glasgow Outcome Scale-Extended (GOSE)^28^,^29^.

Some limitations must be noted in this present study. First, one included study excluded female patients as a result of side effects on the menstrual cycle^12^. Second, due to the lack of available data, we did not analyze other clinical outcomes except mortality and good recovery. It was unknown whether progesterone promoted the recovery of motor and sensory skills. Finally, the follow-up was short-term and varied across the studies. Thus, an appropriate dosage and a long-term follow-up may be necessary to further investigate the efficacy of progesterone in the treatment of acute TBI.

In conclusion, the pooled data did not support the idea that progesterone was superior to placebo in the treatment of acute TBI. Progesterone may be not effective in lowering the incidence of death or vegetative state in patients with acute TBI.

## Methods

### Search strategy

Our electronic search was conducted in PubMed, Embase, and the Cochrane Library databases until May 10, 2015. The core terms included “progesterone” and “head injury,” “traumatic brain injury,” “TBI,” “random,” and “random*”. There was no language limitation. We also searched Google Scholar and checked the reference lists of the included studies to identify any additional eligible articles.

### Inclusion criteria

Studies were included if they met the following criteria: (1) adults (older than 18 years) with a diagnosis of acute TBI, (2) progesterone compared with placebo (or no progesterone), and (3) randomized controlled trials. Duplicate articles, reviews, case reports, and studies without extractable data were excluded.

### Data extraction and outcome measures

Two authors (CL and HQH) independently extracted the following data from each included study in the standard form: (1) study characteristics (author’s name, date of publication, study design, sample size), (2) characteristics of participants (age and gender), (3) interventions (administration, duration, and dosage), and (4) outcomes (GOS and adverse events). Any discrepancies were discussed and resolved by the research team when necessary. The efficacy outcome was assessed with death or vegetative state (GOS = 1 or 2) and good recovery (GOS = 5) at the 6 months after TBI or end of the follow-up period. Adverse events included pneumonia and sepsis.

### Quality assessment

The eligible studies were evaluated according to the Cochrane Collaboration’s tool^30^. The domains were as follow: selection bias (random method and allocation concealment), performance and detection bias (blinding of participants, personnel and outcome assessment), attrition bias (incomplete outcome data), and reporting bias (selective reporting).

### Statistical analysis

The data were analyzed with the Cochrane Review Manager 5.3 and STATA 11.0 software according to the preferred reporting items for systematic reviews and meta-analysis (PRISMA) statement^31^,^32^. Risk ratios (RR) were calculated and pooled with a 95% confidence interval (CI) for dichotomous variables. The heterogeneity was estimated using the I^2^ test, which was considered to be low heterogeneity when I^2^ ≤ 25%. A fixed-effects random effects model was used if the I^2^ was ≤25%. Otherwise, a random effects model was applied. We used the funnel plot and Eger’s test to assess potential publication bias^33^.

## Additional Information

**How to cite this article**: Lin, C. *et al.* Efficacy of progesterone for moderate to severe traumatic brain injury: a meta-analysis of randomized clinical trials. *Sci. Rep.* **5**, 13442; doi: 10.1038/srep13442 (2015).

## Figures and Tables

**Table 1:** Basic characteristics of studies included in the meta-analysis.

**Study/Year**	**Trial design**	**No. of Patients**	**Age (y)**	**Male (%)**	**GCS on admission**	**Treatment**	**Follow-up (M)**
Skolnick 2014	RCT	1179	16 to 70	927(78.63)	4 to 8	Intravenously 0.71 mg/kg for the first hour, then 0.50 mg/kg per hour for 119 hours	6
Shakeri 2013	RCT	76	18 to 60	76(100.00)	3 to 8	orally 1 mg/kg every 12 hours for 5 days	3
Xiao 2008	RCT	159	18 to 65	115(72.33)	≤8	intravenously 1.0 mg/kg every 12 hours for 5 days	6
Wright 2007	RCT	100	old than 18	71(71.00)	4 to 12	intravenously 0.71 mg/kg for the first hour, 0.5 mg/kg per hour for the next 11 hours, then 0.5 mg/kg per hour every 12 hours for 60 hours	1
Wright 2014	RCT	882	17 to 94	650(73.70)	4 to 12	intravenously 0.71 mg/kg for the first hour, then 0.5 mg/kg for 71 hours, then 0.125 mg/kg per hour every 8 hours for 96 hours	6
Xiao 2007	RCT	56	15 to 65	33(58.93)	5 to 8	Intramuscularly 80 mg every 12 hours for 5 days	3
Aminmansour 2012	RCT	40	29.73*	28(70.00)	≤8	intramuscularly 1 mg/kg of progesterone every 12 hours for 5 days	3

RCT, randomized controlled trial; GCS, Glasgow Coma Scale; Y, year; M, month; *mean age.

**Table 2:** Risk of bias of the articles included in the meta-analysis.

**Study, year**	**Randomization method**	**Allocation concealment**	**Data collection blinded**	**Incomplete outcome data**	**Selective reporting**
Skolnick 2014	Low risk	Low risk	Low risk	Low risk	Low risk
Shakeri 2013	Low risk	Unclear risk	High risk	Unclear risk	Low risk
Xiao 2008	Low risk	Low risk	Low risk	Low risk	Unclear risk
Wright 2007	Low risk	Low risk	Low risk	Low risk	Low risk
Wright 2014	Low risk	Low risk	Low risk	Low risk	Low risk
Xiao 2007	Low risk	Unclear risk	Unclear risk	Unclear risk	Unclear risk
Aminmansour 2012	Low risk	Low risk	Unclear risk	Low risk	Low risk

**Table 3:** Subgroup analysis for RCTs evaluating the efficacy in reducing death or vegetative state of patients with acute TBI.

	**N**	**RR**	**95% CI**	**Heterogeneity test(I^2^)**
Date of publication				
Before 2010	3	0.67	0.47, 0.94	0%
After 2010	4	0.99	0.80, 1.23	38%
Sample size				
≤100	4	0.70	0.50, 0.96	0%
>100	3	0.99	0.77, 1.28	56%
Follow-up				
1 m	1	0.43	0.18, 0.99	—
3 m	3	0.77	0.54, 1.10	0%
6 m	3	0.99	0.77, 1.28	56%
Administration				
Intravenously	4	0.90	0.67, 1.22	65%
Intramuscularly	2	0.67	0.37, 1.19	0%
Orally	1	0.71	0.39, 1.27	—

m, month; N, number of studies; CI: confidence interval; RR: risk ratios.
